# Hexokinase 2-mediated glycolysis supports inflammatory responses to *Porphyromonas gingivalis* in gingival fibroblasts

**DOI:** 10.1186/s12903-023-02807-4

**Published:** 2023-02-15

**Authors:** Wenqi Su, Jingwen Li, Lishan Jiang, Lang Lei, Houxuan Li

**Affiliations:** 1 Department of Periodontics, Nanjing Stomatological Hospital, Medical School of Nanjing University, #30 Zhongyang Road, Nanjing, 210008 Jiangsu China; 2 Central Laboratory of Stomatology, Nanjing Stomatological Hospital, Medical School of Nanjing University, Nanjing, China; 3 Department of Orthodontics, Medical School of Nanjing University, Nanjing Stomatological Hospital, Nanjing, China

**Keywords:** Hexokinase 2, Gingival fibroblasts, *Porphyromonas gingivalis*, Glycolysis, Periodontitis

## Abstract

**Background:**

When infected with *Porphyromonas gingivalis*, gingival fibroblasts undergo metabolic reprogramming, and rely on aerobic glycolysis rather than oxidative phosphorylation for rapid energy replenishment. Hexokinases (HKs) are catalysts for glucose metabolism, and HK2 constitutes the major HK inducible isoform. The objective of this study is to determine whether HK2-mediated glycolysis promotes inflammatory responses in inflamed gingiva.

**Methods:**

Levels of glycolysis-related genes were assessed in normal and inflamed gingiva. Human gingival fibroblasts were harvested and infected with *Porphyromonas gingivalis* in order to mimic periodontal inflammation. 2-deoxy-d-glucose, an analogue of glucose, was used to block HK2-mediated glycolysis, while small interfering RNA was used to knock down HK2 expression. The mRNA and protein levels of genes were analyzed by real-time quantitative PCR and western blotting, respectively. HK2 activity and lactate production were assessed by ELISA. Cell proliferation was assessed by confocal microscopy. The generation of reactive oxygen species was assessed by flow cytometry.

**Results:**

Elevated expression of HK2 and 6-phosphofructo-2-kinase/fructose-2,6-biphosphatase 3 was observed in the inflamed gingiva. *P. gingivalis* infection was shown to promote glycolysis in human gingival fibroblasts, as evidenced by increased gene transcription of HK2 and 6-phosphofructo-2-kinase/fructose-2,6-biphosphatase 3, cell glucose consumption, and HK2 activity. Inhibition and knockdown of HK2 resulted in reduced cytokine production, cell proliferation, and reactive oxygen species generation. Furthermore, *P. gingivalis* infection activated the hypoxia-inducible factor-1α signaling pathway, thus promoting HK2-mediated glycolysis and proinflammatory responses.

**Conclusions:**

HK2-mediated glycolysis promotes inflammatory responses in gingival tissues, and therefore glycolysis can be targeted in order to inhibit the progression of periodontal inflammation.

## Introduction

Periodontitis is a chronic inflammatory disease with an overall prevalence of 45–50%; the most severe form of periodontitis affects 11.2% of the world’s population and is the sixth most common human disease [1]. Periodontitis is characterized by microbial-associated, host-mediated inflammation of the gingiva, which is associated with clinical attachment loss, gingival bleeding on probing, and resorption of alveolar bone tissue [2]. Despite the human body’s defense mechanisms against microbes, which can involve the utilization of epithelial cells and defense cells (such as neutrophils and monocytes), bacteria such as *Porphyromonas gingivalis* (*P. gingivalis*) can penetrate the epithelium and infect human gingival fibroblasts [3].

In addition to matrix synthesis and degradation, human gingival fibroblasts can also secrete chemokines that recruit defense cells, such as neutrophils, monocytes, and macrophages, following the recognition of the invading bacteria [4]. Furthermore, human gingival fibroblasts play immunomodulatory roles in the body’s innate response to pathogens by enhancing the phagocytic capability of macrophages [5]. Human gingival fibroblasts are naturally equipped with pattern recognition receptors on their membrane [6]; after the recognition of invading bacteria, they can dynamically adapt their metabolism in order to address microbial invasion.

Sufficient energy production is necessary for resistance against bacterial invasion [7]. The generation of adenosine triphosphate (ATP) can vary according to cell type and the availability of oxygen [8]. The principal sources of energy utilized are carbohydrates, particularly glucose, which can be metabolized by two disparate pathways, specifically, oxidative phosphorylation and anaerobic glycolysis [9]. Although oxidative phosphorylation is energy-efficient, cancer cells tend to favor glycolysis even in an atmosphere with a normal oxygen level. This is referred to as the Warburg effect [10]. Glycolysis, the initial step in the breakdown of glucose for energy production, is mediated by several key enzymes, such as hexokinase 2 (HK2) and 6-phosphofructo-2-kinase/fructose-2,6-biphosphatase 3 (PFKFB3) [11]. HK2 phosphorylates glucose into glucose 6-phosphate (G6P) and acts as a pivotal regulator in glycolysis and cell survival [12].

Previous studies have investigated the pathogenesis of several inflammatory diseases, including rheumatoid arthritis and Alzheimer’s disease [13, 14]. However, there has not been sufficient investigation to determine whether human gingival fibroblasts can undergo metabolic reprogramming from oxidative phosphorylation to glycolysis, and further clarification is needed in order to better understand the regulatory role of metabolic changes in human gingival fibroblasts. Because of its fundamental role in promoting glycolysis and maintaining inflammatory responses, HK2 has become a therapeutic target in the treatment of several diseases. Yuan et al. found that KLF14 caused a reduction in macrophage glycolysis and in inflammatory cytokine secretion by inhibiting HK2 transcription in a mouse anti-sepsis model [15]. It has also been shown that HK2 is a key mediator in the induction of chemotherapy resistance in colon cancer by B7-H3 [16]. However, the role of HK2 and HK2-mediated glycolysis in inflammatory human gingival fibroblasts remains largely unknown.

It has been demonstrated that patients with periodontitis show distinct metabolites in the saliva and gingival crevicular fluid [17], and we recently reported that *P. gingivalis* has been shown to trigger profound metabolic changes in human gingival fibroblasts [18]. Therefore, we inferred that human gingival fibroblasts can transfer their glucose metabolism to glycolysis in response to *P. gingivalis* infection. The principal objective of this study was to explore the potential role of HK2-mediated glycolysis in *P. gingivalis*-stimulated human gingival fibroblasts and further investigate the possible mechanisms underlying the activation of HK2-mediated glycolysis.

## Materials and methods

### Study population and collection of tissue samples

The protocol for collecting normal and diseased gingival samples was approved by the Medical Ethics Committee of Nanjing Stomatological Hospital, Medical School of Nanjing University, and the ethics approval number was 2016NL-010(KS). Normal gingival tissues (n = 8) and inflamed gingival tissues (n = 6) were obtained as described previously [19]. Patients with the following histories were excluded: (I) patients with systemic diseases such as hypertension, diabetes, cardiovascular system diseases, and acquired immune deficiency syndrome; (II) patients being treated with immunosuppressive agents or glucocorticoid therapy; (III) patients who were pregnant or lactating; (IV) patients who smoke; (V) patients with other oral diseases, such as caries, flings or crowns affecting the periodontal state at any sampling site; and (VI) patients who had received periodontal treatment or taken medicine that would affect the condition of the periodontal tissue or their immune systems in the past 6 months.

For the healthy group, gingival tissues were harvested during the crown lengthening surgery or gingivectomy upon exposure of impacted teeth during orthodontic treatment; all the sampling sites displayed no gingival redness and swelling, negative bleeding on probing, probing depth less than 3 mm, and no attachment loss. Gingival tissue was taken from the buccal side of the tooth crown, and gingival tissue with a size of 0.5 cm × 0.3 cm was cut through an internal oblique incision. The inflamed gingival tissues were obtained from patients with severe periodontitis, which belonged to Grade C periodontitis stage III/IV according to the new international classification of periodontal disease and peri-implant disease proposed in 2017 [2]. Samples of inflamed gingival tissue were taken during the extraction of hopeless teeth with vertical, anterior–posterior, and buccal-lingual mobility from patients with advanced periodontitis; sampling sites in the periodontitis group had severe alveolar bone loss (above 2/3), increased probing depths (> 8 mm) and positive bleeding on probing (BOP). In addition, gingival tissue with a size of 0.5 cm × 0.3 cm was cut through an internal oblique incision. The demographic information of all contributors is presented in Table 1.

**Table 1:** Demographic and clinical characteristics of donors

	Normal group	Inflamed group	*p*
Age (year)	33.73 ± 3.727	35.83 ± 2.212	0.6497
Sex			
Male	3	2	–
Female	5	4	–
PD (mm)	2.688 ± 0.2489	8.833 ± 0.5426	< 0.0001**

Data are as mean ± SD or n

**p < 0.01, compared with the normal group

### Cell culture

All primary human gingival fibroblasts from three donors with healthy periodontal status between 18 and 30 years of age were cultured using explant techniques as described [20]. The cells were cultured in a T-25 tissue culture flask (Corning, USA) with 20% fetal bovine serum (Sciencell, USA) and 1% penicillin/streptomycin solution (NCM Biotech, China) and incubated in 5% CO_2_/95% air at 37 °C in an incubator. After 3 days, 3 ml of fresh medium was added. On day 14, cells that adhered to the flask were trypsinized and counted. Cells of 3 to 6 generations were used in the experiment.

### Bacterial strain and culture

*P. gingivalis* ATCC 33,277 was cultured in the brain heart infusion (BHI) medium with hemin (5 mg/l) and menadione (1 mg/l) in the anaerobic condition (85% N_2_, 5% H_2_, and 10% CO_2_). The concentration of bacteria was read by the spectrophotometer at 600 nm.

### Immunohistochemistry

The gingival tissue samples were soaked in 4% paraformaldehyde for 24 h embedded in the wax and cut to 4 μm in thickness. After regular deparaffinization, rehydration, and antigen retrieval with heated citrate buffer (pH 6.0), the sections were incubated with anti-PFKFB3 (1:50; ab181861, Abcam, US), and anti-HK2 (1:500; ab209847, Abcam, US), overnight at 4 °C. Then, the slides were washed three times with phosphate buffer saline (PBS) and incubated with secondary antibodies (MaxVision, China) at room temperature for 30 min. Diaminobenzidine (DAKO, USA) was used as a chromogenic agent to detect antibody binding. The percentage of positive cells in ten random visual fields for healthy and periodontitis tissues, as counted with the software immunohistochemistry (IHC) profiler by two independent observers who were blinded to the samples, respectively.

### Isolation of RNA and quantitative PCR

Total RNA was extracted from human gingival fibroblasts using an RNAprep pure Cell /Bacteria Kit (TIANGEN, China) and measured using a Nanodrop (Thermo Fisher Scientific, USA). Reverse transcription was carried out immediately using a PrimescriptTM RT reagent kit (Vazyme, China) according to the manufacturer’s protocol. Real-time PCR was conducted in triplicates using SYBR Green Master MIX (ABI, USA). Genscript (Genscript, China) synthesized the sequences of the primers for quantitative real-time PCR (qPCR). The sequences of primers were present in Table 2. Relative quantification was achieved using the comparative 2^−△△Ct^ method.

**Table 2:** The primer sequences used for real-time PCR

Genes	Sequence (5′–3′)	Sequence (3′–5′)
PFKFB3	TTGGCGTCCCCACAAAAGT	AGTTGTAGGAGCTGTACTGCTT
HK2	GAGCCACCACTCACCCTACT	CCAGGCATTCGGCAATGTG
IL-6	GAAAGCAGCAAAGAGGCACT	TTTCACCAGGCAAGTCTCCT
MCP-1	CAGCCAGATGCAATCAATGCC	TGGAATCCTGAACCCACTTCT-5
β-actin	GTGGGGCGCCCCAGGCACCA	CGGTTGGCCTTGGGGTTCAGGGGGG

### Western blot analysis

Western blotting was performed as we described before [21]. Human gingival fibroblasts were lysed with ice-cold RIPA buffer (Beyotime Biotechnology, China) and the concentration of protein was determined using a Nano drop (Thermo Fisher Scientific, USA). Proteins were separated by sodium dodecyl sulfate–polyacrylamide gel electrophoresis (SDS-PAGE) (Smart-Lifesciences, China), transferred to polyvinylidene fluoride (PVDF) membrane (Millipore, USA), and blocked with quick block™ blocking buffer (Beyotime, China). The membranes were blocked with 5% bovine albumin and incubated with primary antibodies to PFKFB3 (1:1000; ab181861, Abcam, US), HK2 (1:1000; ab209847, Abcam, US), hypoxia-inducible factor (HIF)-1α (1:1000; ab2185, Abcam), proline hydroxylase (PHD)2 (1:800; D31E11, CST, Germany), β-actin (1:1000; 66,009-l-lg, Proteintech, China). Followed by secondary antibodies (Thermo Fisher Scientific, USA). Protein bands were detected with Image Quant LAS 4000. In order to reduce non-specific protein expression during the experiment, the membrane was trimmed according to the protein size range provided in the antibody instructions prior to hybridization with the antibody. Each experiment was repeated three times.

### Glucose consumption and lactate production and HK2 activity determination

Human gingival fibroblasts were stimulated with different agents for 24 h and the supernatant was collected, and cells were washed with PBS. The level of glucose and lactate was detected with enzyme-linked immunosorbent assay (ELISA) kits (Keshun Science and Technology Corporation, Shanghai, China). HK2 activity was detected with enzyme-linked immunosorbent assay (ELISA) kits (Jin Yibai, China) according to the manufacturer’s instructions. When the cell concentration reached about 1 million/l, cells were repeatedly frozen and thawed to extract intracellular substances, and intracellular components were released. The cell suspension was centrifuged at 2000–3000 rpm per min for 20 min and was collected carefully. The optical density of each well was determined immediately using a Spectra Max M3 (Molecular Devices, USA) at 450 nm.

### Reactive oxygen species detection by flow cytometry

Intracellular reactive oxygen species (ROS) in human gingival fibroblasts were detected using the reactive oxygen species assay kit (Beyotime, China). The reagent uses the fluorescent probe DCFH-DA to enter cells and becomes highly fluorescent after being oxidized by reactive oxygen species. Human gingival fibroblasts were seeded at a density of 2 × 10^5^ cells in a 6-well cell culture plate and treated with *P.gingivalis* for 24 h. Cells were incubated with 10 μM DCFH-DA reagent for 30 min at 37 °C and washed with PBS. Subsequently, cells were scraped in the PBS, before being transferred to the polypropylene FACS tubes. Cells were centrifuged at 2000 rpm per min for 3 min. Cells were washed in the PBS and centrifuged two further times, and were finally suspended in the PBS (300 μl). The cultures were analyzed by flow cytometry (BD Bioscience, USA). The data were analyzed with FlowJo v.9.5.2 software (Tree Star).

### Small interference RNA transfection

Small interfering RNA (siRNA) targeting HK2, and scramble siRNAs were purchased from PPL (Public Protein/Plasmid Library, China). The cells were seeded in 6-well plates. When reaching 60–70% confluency, cells were transfected with scramble (50 nM) or HK2 siRNA (50 nM) for 6 h using Lipofectamine 3000 transfection reagent (Thermo Fisher, USA). Afterward, the cells were cultured in the DMEM medium for further treatments. The efficacy of knockdown was analyzed by qPCR 48 h and Western blot 72 h after the transfection.

### Enzyme-linked immunosorbent assay

Human gingival fibroblasts were stimulated with the indicated stimuli for 24 h, the supernatant was collected for the detection of interleukin (IL)-6, and monocyte chemoattractant protein (MCP)-1 with ELISA kits (MultiSciences, China). The optical density was read using a Spectra Max M3 (Molecular Devices, USA).

### Statistical analysis

The Shapiro–Wilk test and F test were used to analyze the normality and homogeneity of variance of the data. Data were evaluated by analysis of variance (ANOVA) and the Dunnett multiple-comparison test was used. Where appropriate (comparison of two groups only), a student’s t-test was performed, and *p* < 0.05 was taken as the level of significance. The data was presented by Graph Pad Prism 7.00 (Graph Pad Software Inc, LaJolla, CA, USA). All data were shown as mean ± standard deviation. Statistical values, including a number of replicates (n), can be found in the figure legends.

## Results

### Expression of glycolysis-related genes was elevated in inflamed periodontal tissues

**Fig. 1:**
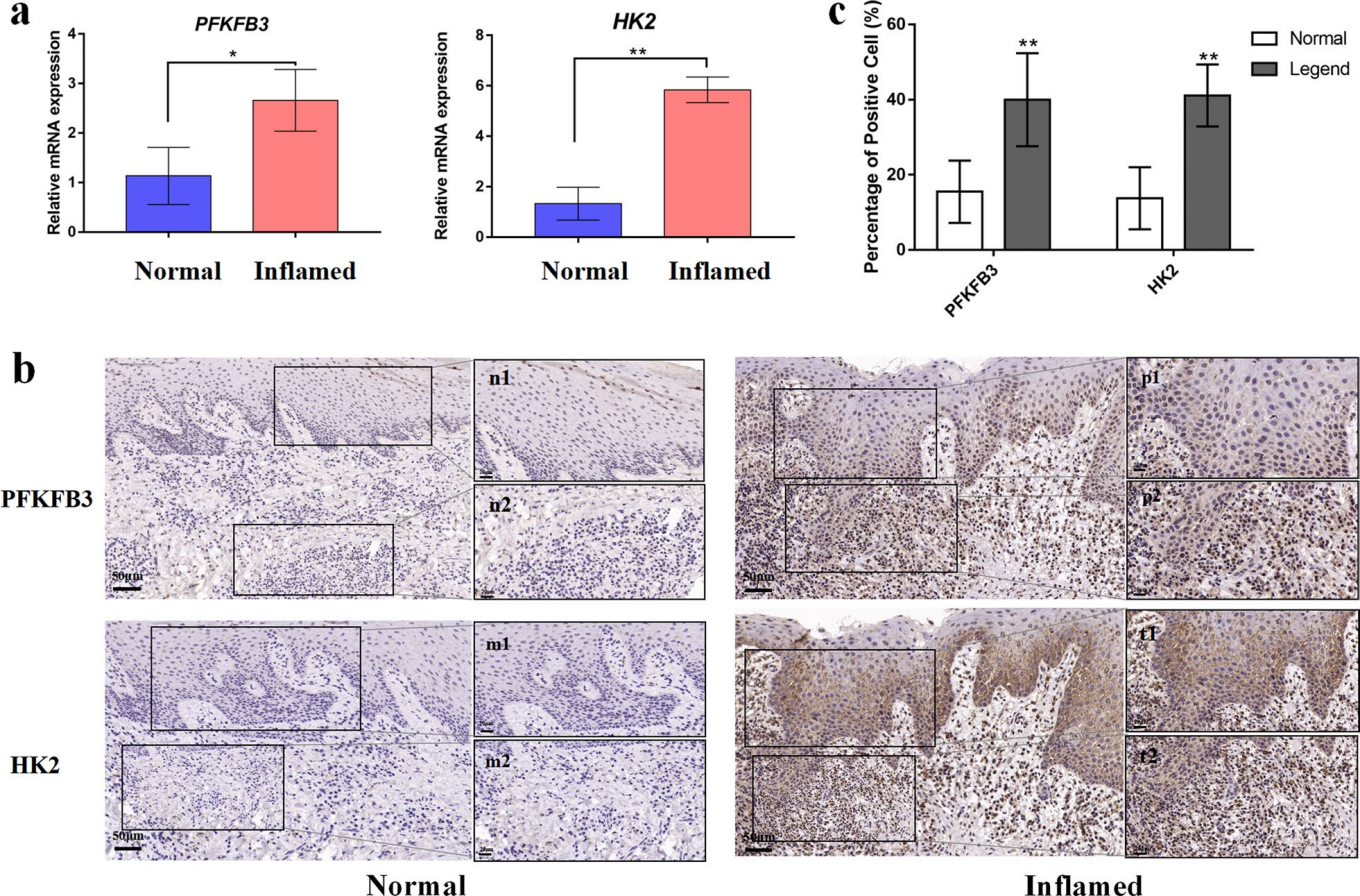
Increased expression of glycolysis-related genes in inflamed gingival tissues. **a** Gene transcription of HK2 and PFKFB3 in normal (n = 8) and inflamed (n = 6) gingival tissues was measured by qPCR. **b** Protein expression of HK2 and PFKFB3 in normal (n = 6) and inflamed (n = 6) gingival tissues was determined by immunohistochemistry staining. The figures (n1/2, m1/2, p1/2 and t1/2) show higher magnifications of the same sections in the indicated area. The scale bar represents 50 µm. **c** Quantification of positive cells from the immunohistochemistry staining. β-actin was used as an internal reference. The data were analyzed by Student’s t-test, and values are presented as the mean ± SD; *, *p* < 0.05 and **, *p* < 0.01, compared with the normal group. Figures n1/2 and m1/2 represent the expression of PFKFB3 and HK2 in the epithelial and connective tissue of the normal control group at higher magnification. Figures p1/2 and t1/2 represent the expression of PFKFB3 and HK2 in the epithelial tissue and connective tissue of the inflamed group at higher magnification

We first explored the gene transcription of key glycolysis-related enzymes in the gingiva. The demographic information on all of the 14 subjects is shown in Table 1. There was no statistically significant difference in age between the 8 healthy subjects and the 6 subjects with periodontitis. Compared to the healthy control, increases of nearly four and one fold were observed in the mRNA transcription of HK2 and PFKFB3, respectively (Fig. 1a). Low levels of PFKFB3 and HK2 were observed in the healthy gingiva, whereas elevated levels of PFKFB3 and HK2 were observed in the inflamed gingiva (Fig. 1b), indicating an elevation of glycolysis in the inflamed gingiva.

### *P. gingivalis* enhanced HK2-mediated glycolysis in human gingival fibroblasts

**Fig. 2:**
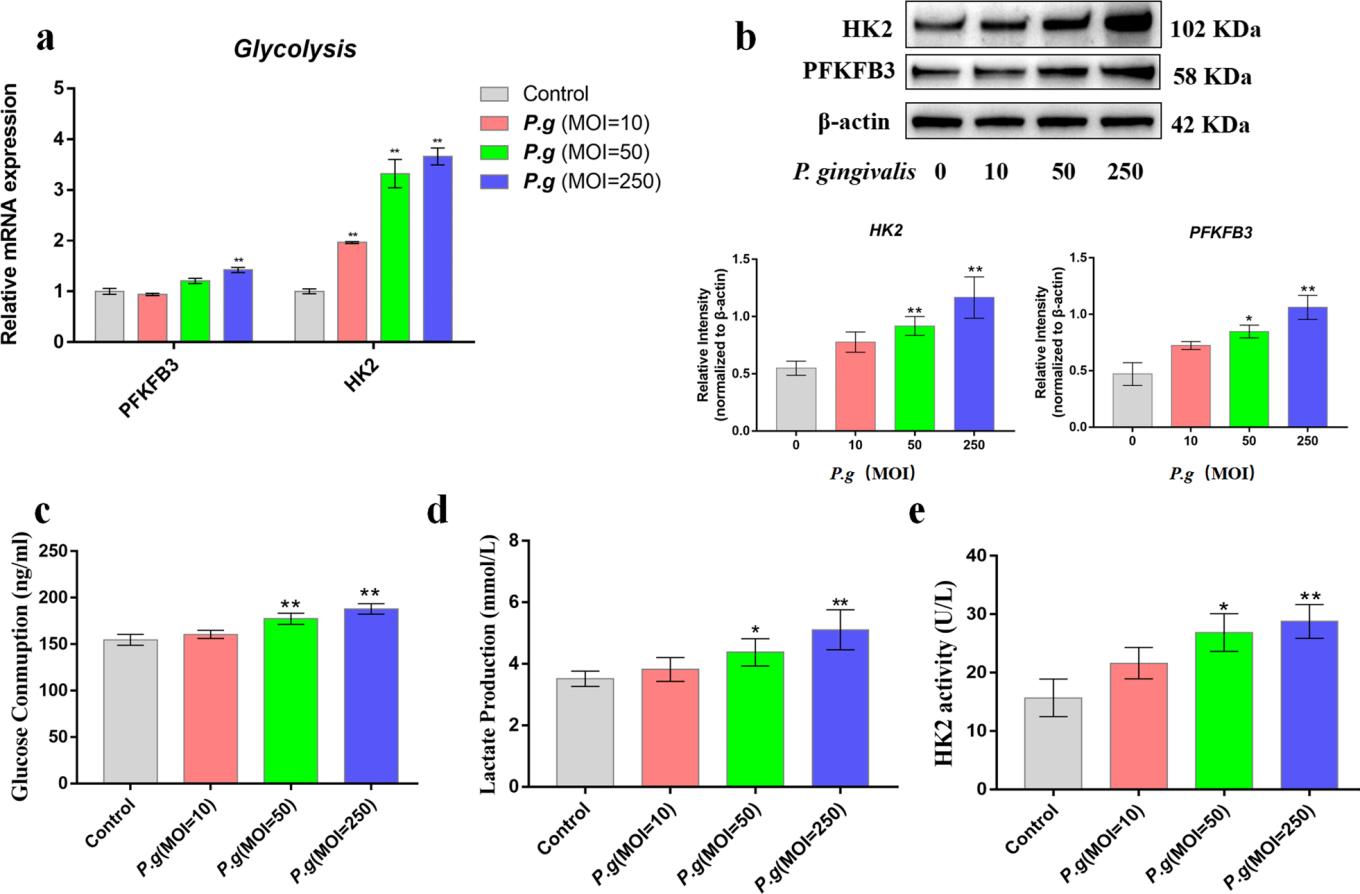
*P. gingivalis* infection promotes HK2-mediated glycolysis in human gingival fibroblasts. Human gingival fibroblasts were infected with *P. gingivalis* (MOI = 10, 50, or 250). **a** Gene transcription was assessed at 4 h by qPCR. **b** Protein expression was analyzed by western blotting at 24 h. **c**–**e** HK2 activity; glucose consumption and lactate production were monitored using enzymatic detection kits. β-actin was adopted as an internal reference. The data were analyzed by ANOVA and a Dunnett multiple-comparison test, and values are presented as the mean ± SD; *, *p* < 0.05 and **, *p* < 0.01, compared with the control group

*P. gingivalis*, an anaerobic periodontal pathogen, can invade the junctional epithelium and then trigger inflammatory responses in gingival fibroblasts [22]. In order to mimic the inflammatory status in the periodontal niche, human gingival fibroblasts, being the predominant resident cells in the gingiva, were infected with *P. gingivalis* at multiplicities of infection (MOIs) of 10, 50, and 250. Gene transcription of HK2 and PFKFB3 was significantly increased in the bacteria-infected human gingival fibroblasts in a concentration-dependent manner (Fig. 2a); protein levels of the two glycolytic genes were significantly upregulated by *P. gingivalis* stimulation in human gingival fibroblasts (Fig. 2b). In addition, glucose consumption was elevated after infection with *P. gingivalis* (Fig. 2c), whereas the level of lactate in the culture supernatant was not significantly increased after bacterial infection (Fig. 2d). Moreover, HK2 activity, an indicator of glycolysis, was increased after *P. gingivalis* infection at MOIs of 50 and 250 (Fig. 2e). These data demonstrate that *P. gingivalis* stimulation promotes glycolysis in human gingival fibroblasts.

### Glycolysis promoted human gingival fibroblast inflammatory responses to *P. gingivalis*

**Fig. 3:**
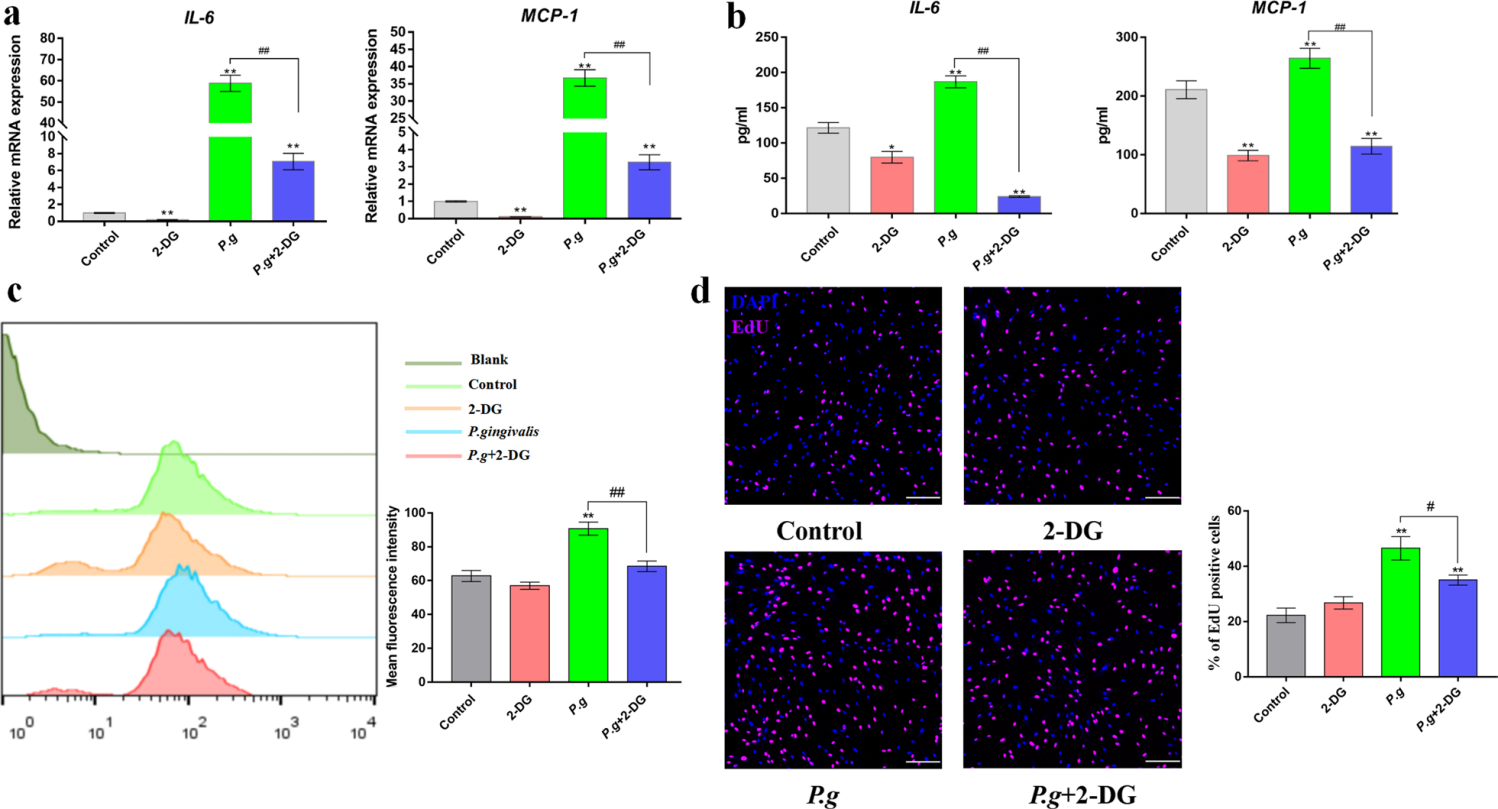
HK2-mediated glycolysis promotes inflammatory responses to *P. gingivalis*. Human gingival fibroblast cells were pretreated with 2-DG (5 mM) for 2 h, followed by *P. gingivalis* infection (MOI = 50). **a** Gene transcription was assessed by qPCR at 4 h. **b** Cytokine expression in the culture supernatants was measured by ELISA at 24 h. **c** Intracellular reactive oxygen species (ROS) were assessed by flow cytometry at 24 h. **d** The cell proliferation rate was quantified by EdU incorporation at 24 h. The scale bar represents 100µm. β-actin was used as an internal reference. The data were analyzed by ANOVA and the Dunnett multiple-comparison test, and values are presented as the mean ± SD; *, *p* < 0.05 and **, *p* < 0.01, compared with the control group; #, *p* < 0.05 and ##, *p* < 0.01, compared with the *P. g* group

In order to investigate the role of glycolysis in the inflammatory responses to *P. gingivalis* in human gingival fibroblasts, we utilized 2-deoxy-d-glucose (2-DG), a synthetic glucose analogue that can competitively inhibit glucose uptake [23], and thus block glycolysis. 2-DG was administered for 2 h before bacterial infection (MOI = 50). Pretreatment with 2-DG (5 mM) for 2 h significantly reduced the gene transcription of IL-6 and MCP-1 in *P. gingivalis*-infected human gingival fibroblasts (Fig. 3a). The same effect was observed with the protein expression of IL-6 and MCP-1 in the culture supernatants (Fig. 3b). Because the generation of reactive oxygen species is intrinsic to inflammatory responses, and because the production of reactive oxygen species can enhance proinflammatory responses by activating the classical nuclear-factor kappa B (NF-κB) signaling pathway [24], we next sought to determine whether 2-DG pretreatment could reduce the production of reactive oxygen species in *P. gingivalis*-infected human gingival fibroblasts. As shown in the flow cytometry data, 2-DG caused a significant decrease in the production of intracellular reactive oxygen species (Fig. 3c). Because the overgrowth of gingival tissues is a common feature in gingivitis and periodontitis, we next investigated the effects of glycolysis on the proliferation of human gingival fibroblasts. Increased cell proliferation was observed in the *P. gingivalis*-infected human gingival fibroblasts after BeyoClick™ EdU staining, whereas 2-DG pretreatment significantly reduced the inflammatory proliferation of *P. gingivalis*-infected human gingival fibroblasts (Fig. 3d).

### HK2 knockdown inhibited human gingival fibroblast inflammatory responses to *P. gingivalis*

**Fig. 4:**
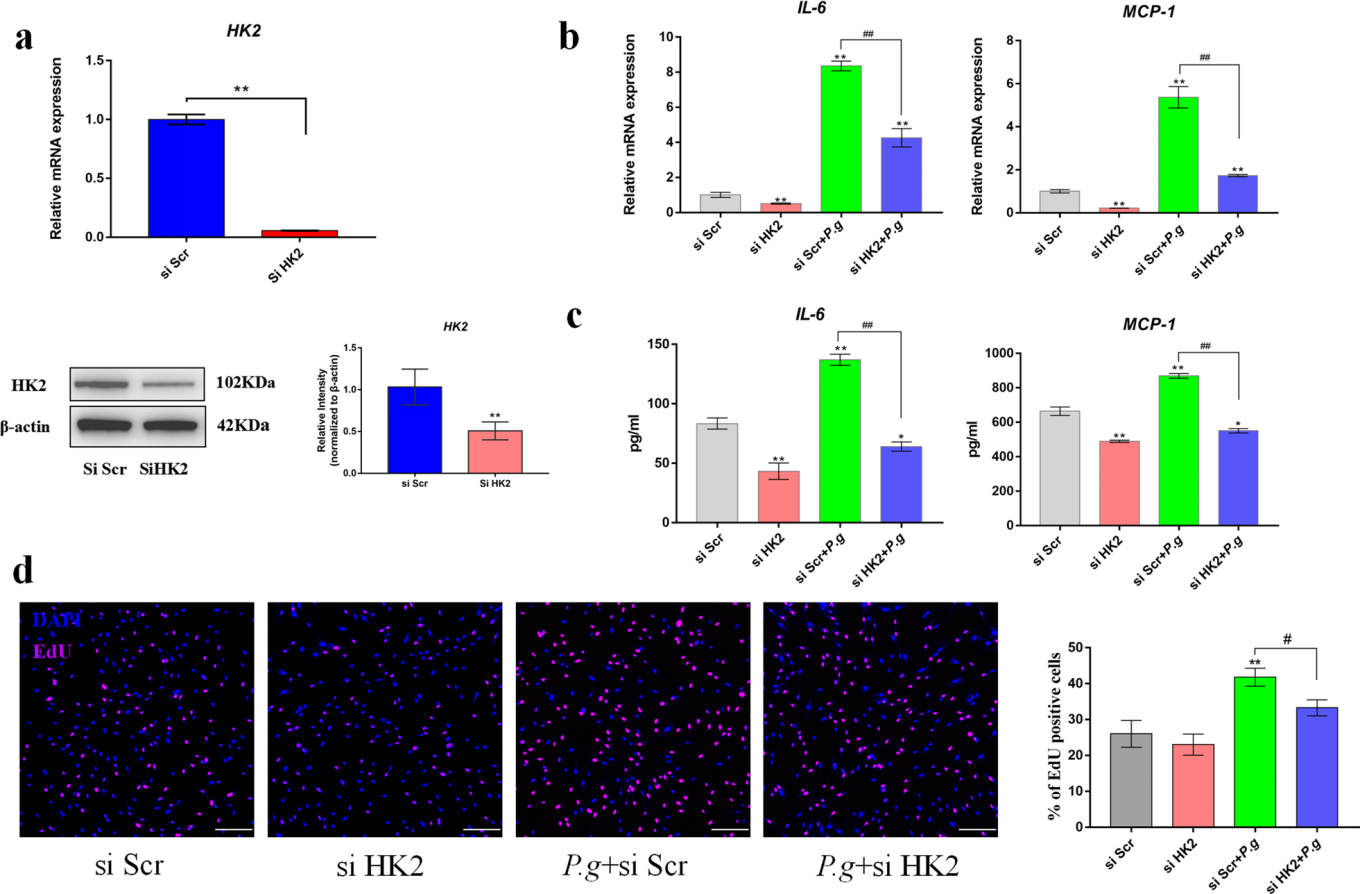
HK2 knockdown reduces inflammatory responses to *P. gingivalis*. Human gingival fibroblasts were transfected with small interfering scrambled control (si Scr) and si HK2 siRNA. **a** The efficiency of HK2 knockdown was determined by qPCR and western blotting at 48 and 72 h, respectively. **b** Gene transcription was assessed by qPCR at 4 h. **c** Protein expression in the culture supernatants was determined by ELISA at 24 h. **d** The proliferation rate was quantified by EdU incorporation at 24 h, and the scale bar represents 100 µm. β-actin was used as an internal reference. The data in (**a**) were analyzed by Student’s t test. The data in (**b**–**d**) were analyzed by ANOVA and the Dunnett multiple-comparison test, and values are presented as the mean ± SD; *, *p* < 0.05 and **, *p* < 0.01, compared with the si Scr group; #, *p* < 0.05 and ##, *p* < 0.01, compared with the *P. g* + si Scr group

After proving that glycolysis promoted inflammatory responses to *P. gingivalis,* we next sought to determine whether HK2 mediates inflammation in human gingival fibroblasts. Hence, siRNA-targeting HK2 was used to further verify the role of HK2-mediated glycolysis in inflammatory responses. Successful knockdown of HK2 was achieved, as was evidenced by gene transcription and protein expression in qPCR and western blotting analyses, respectively (Fig. 4a). Gene transcription of IL-6 was downregulated by HK2 siRNA from eight fold to four fold in *P. gingivalis*-infected human gingival fibroblasts, while transcription of MCP-1 was decreased from nearly five fold to two fold in *P. gingivalis-*infected human gingival fibroblasts after HK2 knockdown (Fig. 4b). Similarly, the knockdown of HK2 contributed to a significant reduction in the protein expression of IL-6 and MCP-1 in the culture supernatants (Fig. 4c). In addition, the knockdown of HK2 caused a significant decrease in the proliferation of human gingival fibroblasts by *P. gingivalis*, as shown by immunofluorescence staining (Fig. 4d). Therefore, all the data indicates that HK2 knockdown suppresses cell inflammatory responses in *P. gingivalis-*infected human gingival fibroblasts.

### HIF-1α activation promoted HK2 expression and inflammatory responses in human gingival fibroblasts

**Fig. 5:**
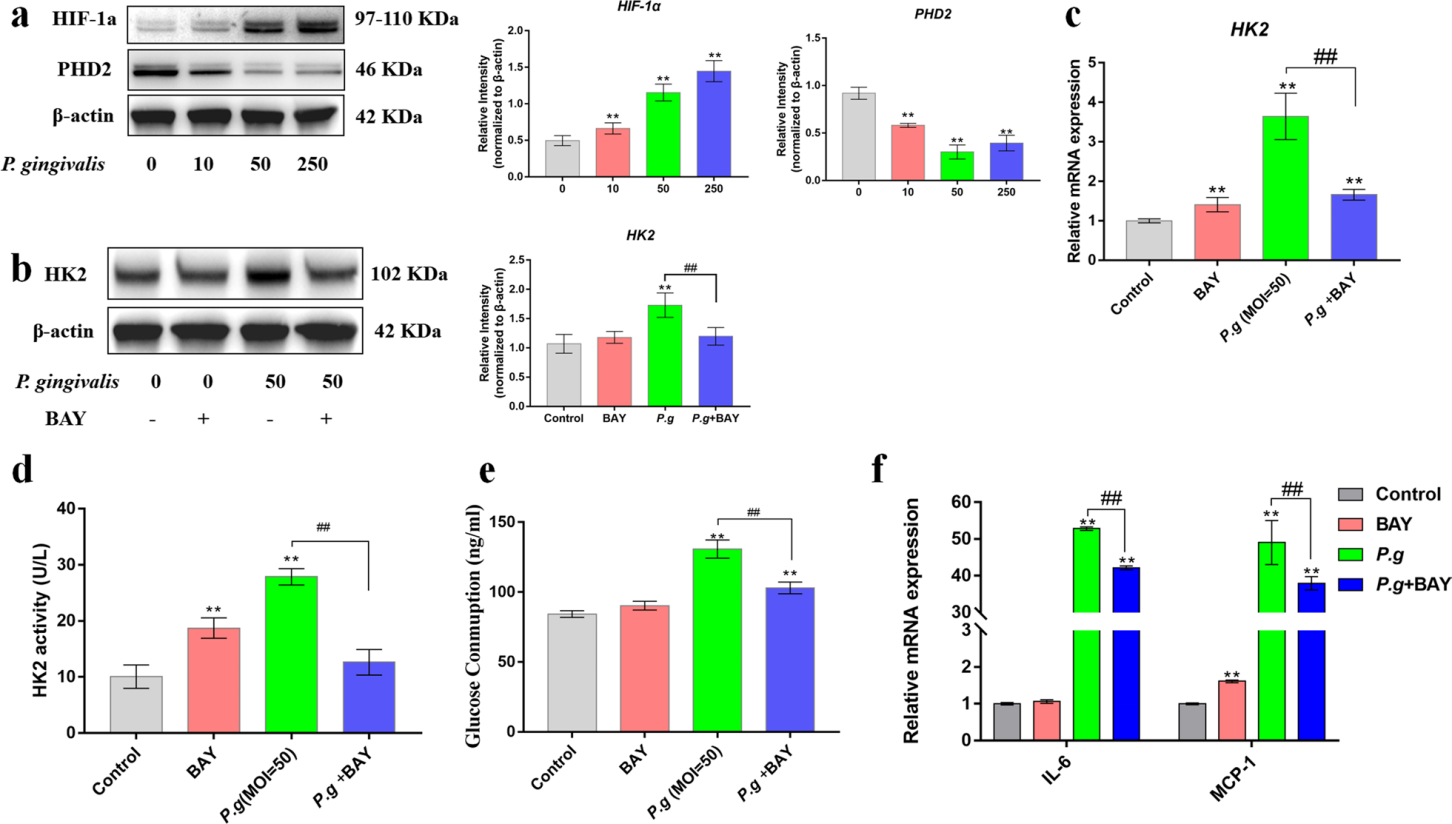
PDH2/HIF-1α signaling pathway activation promoted HK2-mediated glycolysis and inflammatory responses. **a** Protein expression of hypoxia-inducible factor (HIF)-1α and prolyl hydroxylase (PHD)2 was assessed by western blotting after *P. gingivalis* infection (MOI = 10, 50 or 250) for 1 h. Human gingival fibroblasts were treated with HIF-1α inhibitor (10 nM BAY 87–2243) for 2 h before infection with *P. gingivalis* (MOI = 50). **b** Protein expression of HIF-1α and PHD2 was assessed by western blotting at 1 h. **c** Protein expression of HK2 was assessed by western blotting at 24 h. **d**, **e** Glucose consumption and HK2 activity were assessed by enzymatic kits at 24 h. **f** Gene transcription was assessed by qPCR at 4 h. β-actin was used as an internal reference. The data were analyzed by ANOVA and the Dunnett multiple-comparison test, and values are presented as the mean ± SD; *, *p* < 0.05 and **, *p* < 0.01, compared with the control group; #, *p* < 0.05 and ##, *p* < 0.01, compared with the *P. g* group

The transcription factor HIF-1α and its regulator PHD2 play pivotal roles in regulating cell metabolism by regulating downstream gene expression levels [21]. Therefore, we next sought to determine whether the surge in HK2-mediated glycolysis in human gingival fibroblasts after bacterial infection was a result of HIF-1α activation. *P. gingivalis* infection elevated the levels of HIF-1α and reduced the level of PHD2 in human gingival fibroblasts, and this effect was concentration-dependent on the MOI of *P. gingivalis* (Fig. 5a). Subsequently, we treated human gingival fibroblasts with BAY 87–2243 (10 nM), a specific HIF-1α inhibitor, for 2 h. The protein expression of HK2 was decreased in *P. gingivalis*-infected human gingival fibroblasts after blocking HIF-1α (Fig. 5b). In addition, BAY effectively inhibited HK2 transcription, as shown by qPCR (Fig. 5c). BAY treatment significantly reduced HK2 activity and glucose consumption in bacteria-infected human gingival fibroblasts (Fig. 5d,e). Furthermore, the blocking of HIF-1α by BAY treatment significantly reduced gene transcription of the proinflammatory cytokines IL-6 and MCP-1 in human gingival fibroblasts (Fig. 5f). Therefore, these data indicate that HK2-mediated glycolysis is dependent on the activation of the HIF-1α signaling pathway.

## Discussion

Human gingival fibroblasts, the principal components of gingival tissues, participate in the synthesis of collagen fibers, and we recently observed that human gingival fibroblasts can dynamically reprogram cellular metabolism during *P. gingivalis* infection [18]. Our present research further demonstrated that *P. gingivalis* promoted HK2-mediated glycolysis in human gingival fibroblasts, resulting in elevated cytokine expression and cell proliferation; in addition, the elevation in HK2-mediated glycolysis was dependent on the activation of the HIF-1α signaling pathway. This enhancement of glycolysis in human gingival fibroblasts can fundamentally shape the progress of periodontal inflammation. Therefore, HK2-mediated glycolysis may be a potential target for the host-modulated treatment of periodontal disease in the future, through the topical use of HK2 inhibitors.

Along with several sources of energy production, including fatty acids and amino acids, glucose metabolism plays a central role in combatting bacterial infection in monocytes and macrophages [25]. *P. gingivalis* triggers a metabolic change from oxidative phosphorylation to glycolysis in macrophages and periodontal ligament fibroblasts [21, 26]. In this study, elevated expression of HK2 and PFKFB3 was observed in the inflamed gingiva (Fig. 1), indicating enhanced glycolysis in the inflamed gingiva. Moreover, *P. gingivalis* promoted glucose consumption and HK2 activity in human gingival fibroblasts (Fig. 2), which further indicates that HK2-mediated glycolysis plays a role in the inflammatory responses to *P. gingivalis* in the gingiva.

HK plays multiple roles in the mediation of innate immune responses during bacterial infection. HK can act as an intracellular receptor for the recognition of peptidoglycan, the principal component of the cell walls of gram-positive bacteria [11]. Human gingival fibroblasts respond to bacterial infection by producing a variety of chemokines and cytokines, including IL-1, IL-6, and MCP-1 [27]*.* IL-6 induces glycolysis in Raw264.7 macrophages by activating Akt signaling and promoting HK-2 expression and mitochondrial translocation [28]. Enhanced activity of HK2 can trigger profuse production of glucose-6-phosphate, which can trigger inflammasome activation [29]; in addition, IL-6, IL-8, and C-X-C motif chemokine ligand 9 (CXCL9) production was shown to be reduced in LPS-treated macrophages after the inhibition or knockdown of HK2 [30, 31]. In this study, it was further demonstrated that the inhibition and knockdown of HK2 reduced cytokine production, including IL-6 and MCP-1, in human gingival fibroblasts (Figs. 3, 4).

Oxidative stress is central to periodontal tissue damage caused by host-microbe interactions. It can be a direct result of the excessive activity of reactive oxygen species/lack of antioxidants, or it can be an indirect result of the activation and production of redox-sensitive transcription factors [32]. Cellular components of bacterial and inflammatory cytokines cause the recruitment and activation of highly reactive polymorph nuclear cells, which accelerate the production of reactive oxygen species [33]. HK, in combination with mitochondria, participates in glucose metabolism and the regulation of ROS generation [34]. This study found that with *P. gingivalis* infection in human gingival fibroblasts, there was an increase in the expression of inflammatory factors in cells, and an accumulation of reactive oxygen species (Fig. 3). 2-DG, a glucose analogue, is often used to inhibit intracellular glycolysis and produce ATP, an energy-generating molecule that is critical to healthy cell function [35]. Therefore, we sought to determine whether 2-DG could reduce the inflammation level and increase levels of reactive oxygen species after *P. gingivalis* stimulation. It was demonstrated that 2-DG effectively inhibits the increase in reactive oxygen species (Fig. 3), which is consistent with the research results of Zhang et al. [36].

Apart from the explosive production of neutrophils and monocytes in order to eradicate bacteria, resident gingival fibroblasts can alert immune cells and proliferate in order to contain the pathogen infection [37]. Glycolytic metabolic intermediates can fuel anabolic processes in proliferating cells [38]. LPS promotes aerobic glycolysis and enhances collagen synthesis in pulmonary fibroblasts [39]. Additionally, we observed that cell proliferation was increased in *P. gingivalis-*infected human gingival fibroblasts, while the inhibition of glycolysis by 2-DG as well as HK2 knockdown reduced cell proliferation (Figs. 3, 4). Therefore, it can be concluded that the enhanced glycolysis in human gingival fibroblasts upon *P. gingivalis* infection may fuel catabolic events in the inflamed gingiva, which indicates that targeting HK2-mediated glycolysis can help to inhibit the abnormal proliferation of gingival fibroblasts in an inflammatory environment.

TBK1-IKKɛ-Akt signaling is critical to the process of triggering bacteria-induced glycolysis by promoting the association of HK2 with mitochondria [40]. Elevated glycolysis and enhanced HK2 activity in human monocyte-derived dendritic cells after TLR4 ligation are mediated by p38-dependent hypoxia-inducible factor-1α stabilization [30]. HIF-1α has been positively correlated with HK2 expression in breast cancer tissues, and bioinformatics analysis has shown that HIF-1α has a hypoxic response element (HRE) in the upstream promoter region of HK2 [41]. This study also demonstrates that the enhancement of HK2 expression is dependent on HIF-1α stabilization (Fig. 5). Further experiments have not been conducted to explore the mechanism of HIF-1α stabilization; however, we have previously reported that *P. gingivalis* induced the accumulation of succinate, a substrate of succinate dehydrogenase, in the cytosol [21]. Succinate can inhibit 2-oxoglutarate-dependent dioxygenases; including PHDs. High levels of succinate can reduce dioxygenase activity by product inhibition because dioxygenases generate succinate as a product [42]. Therefore, metabolic reprogramming, causing a change from oxidative phosphorylation to glycolysis, can promote HIF-1α signaling pathway activation, which can further enhance glycolysis, in a vicious cycle that promotes proinflammatory responses in human gingival fibroblasts upon bacterial infection.

*P. gingivalis* and *Tannerella forsythia* are highly associated with chronic periodontitis and can be detected in up to 85% of disease sites [43], whereas they are rarely detected or in small numbers in healthy sites. Therefore, in this study, human gingival fibroblasts were infected with *P. gingivalis* in order to mimic the periodontal inflammatory microenvironment in vitro. However, periodontal disease is polymicrobial in nature, rather than a single infection by *P. gingivalis*, and polymicrobial synergy may contribute to periodontal disease [44]. Indeed, the less pathogenic *Fusobacterium nucleatum* and the more pathogenic red-complex bacteria *P. gingivalis* and *Treponema denticola* trigger different responses in human gingival fibroblasts [45], which also exhibit different responses after interferon-γ or IL-4 priming [46]. It is therefore indicated that other periodontal pathogenic bacteria can enhance HK2-mediated glycolysis and support the inflammatory responses to human gingival fibroblasts, albeit at different levels. Further studies are needed to determine whether the difference in HK2-mediated glycolysis underlies the inflammatory responses to various periodontopathogens.

It is important to note that *P. gingivalis* can maintain stable growth in environments with a low oxygen content (3% and 6%), while live *P. gingivalis* can experience more bacterial mortality with prolonged normoxic exposure [47]. Thus, cell-bacteria culture in vitro may not accurately mimic the in vivo environment. Although we presented data showing that inflammatory responses to *P. gingivalis* are inhibited with the inhibition of HK2, an animal model should be utilized in order to determine whether the modulation of HK2 expression can reduce the progression of periodontitis. In this study, as *P. gingivalis* was cultured with cells under normoxic conditions, inflammatory changes were explored over a short time period in order to maintain *P. gingivalis* activity. However, there is a need for the assessment of longer time points in order to explore the regression of the inflammatory response, and there is also a need for in-depth testing of metabolites in specific pathways in order to reveal a more detailed role of metabolism in the enhancement of the inflammatory response of gingival fibroblasts during bacterial infection.

**Fig. 6:**
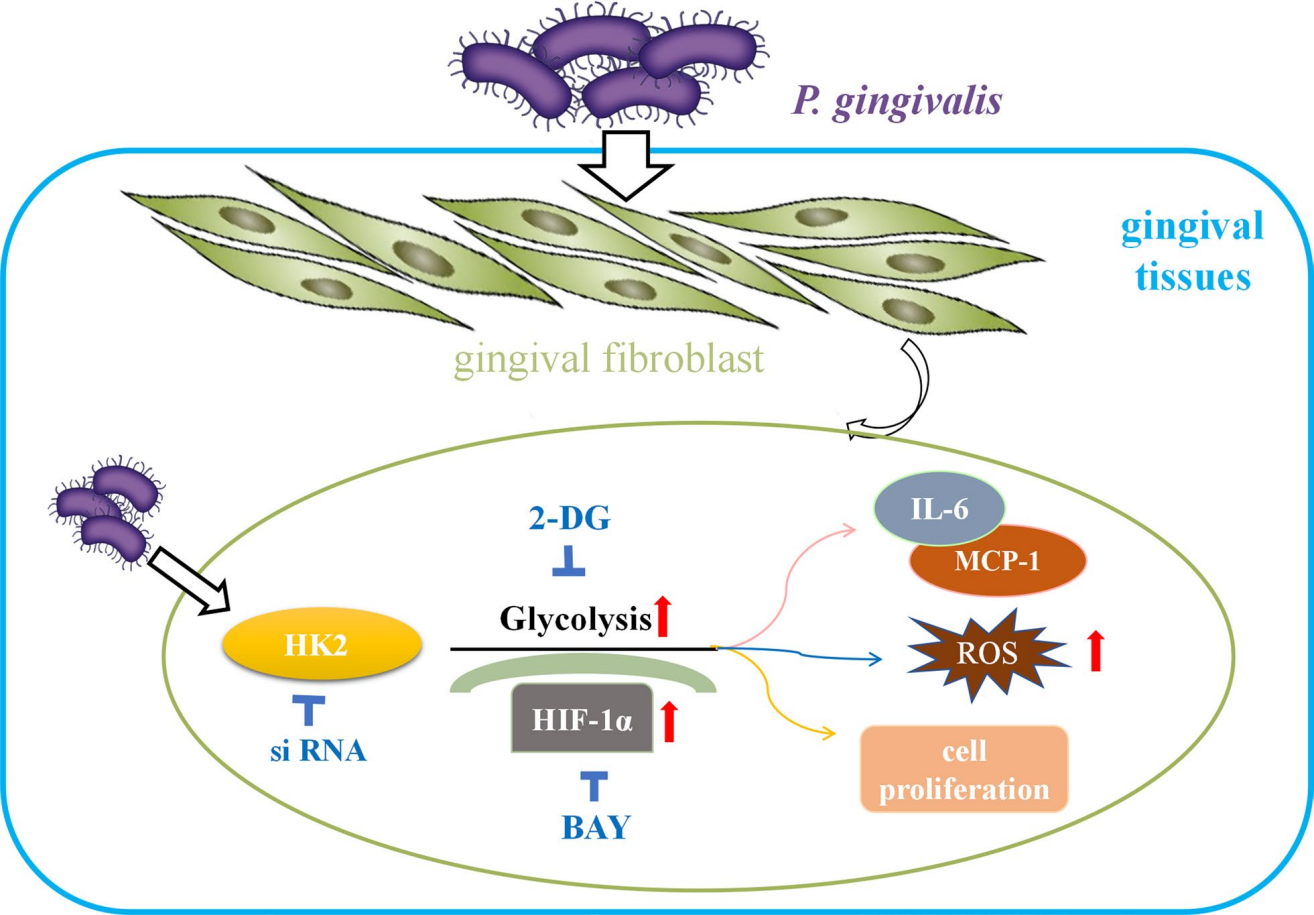
Schematic illustration of the mechanism by which HK2 supports the inflammatory response of gingival fibroblasts to *P. gingivalis*. After infection with *P. gingivalis*, HK2 activity in the gingival fibroblasts was increased, leading to elevated glycolysis, an increase in inflammatory cytokines and reactive oxygen species, and increased cell proliferation. Inhibition of glycolysis or knockdown of HK2 can effectively reduce inflammation and the proliferation of gingival fibroblasts, and reduce the generation of reactive oxygen species

This study demonstrated that *P. gingivalis* infection triggers the stabilization of HIF-1α, which promotes glycolysis, and that HK2-mediated glycolysis enhances the inflammatory responses to *P. gingivalis* infection. We summarize the mechanism by which HK2 plays a critical role in the inflammatory response to *P. gingivalis* in Fig. 6. However, further studies are needed to reveal whether host cells can acquire energy by metabolizing amino acids and fatty acids, as well as to determine whether long-term application of HK2 inhibitors can be beneficial for controlling inflammation in the periodontal niche.

## Data Availability

The datasets used and/or analyzed during the current study are available from the corresponding author upon reasonable request.
